# Knowledge, Perception, and Willingness to Use Artificial Intelligence for Personalized Health Recommendations Among Undergraduate Health Students: A Cross-Sectional Study

**DOI:** 10.7759/cureus.104430

**Published:** 2026-02-28

**Authors:** Munrerah Almulhem, Ameerah Almusailem, Mona Alayesh, Alzahraa Bu Najimah, Hawra Bin Karam, Hala Alsaleem, Haylah Almajhad, Zainab Alessa, Manahil Alfridan

**Affiliations:** 1 Public Health, King Faisal University, Hofuf, SAU; 2 Health Informatics, King Faisal University, Hofuf, SAU

**Keywords:** artificial intelligence, healthcare technology, health students, personalized health, willingness to use ai

## Abstract

Background

Artificial intelligence (AI) is increasingly integrated into healthcare, with uses including personalized health recommendations. Understanding the knowledge, perceptions, and willingness of future healthcare professionals to engage with AI is essential for supporting its effective adoption. This study examined the knowledge, perception, and willingness to use AI among undergraduate students in the health colleges at King Faisal University.

Methods

A descriptive cross-sectional study was conducted, including 417 undergraduate students from 5 health colleges. A structured electronic questionnaire was used to assess students’ knowledge of AI, their perception of its use in healthcare, and their willingness to use AI-based personalized health recommendations. Associations between variables were evaluated using descriptive statistics and logistic regression analysis.

Results

Among the 417 participants, 67.87% had a high level of knowledge about AI, 57.07% had a positive perception, and 74.82% reported willingness to use AI for personalized health recommendations. Logistic regression analysis showed that students with high knowledge were more likely to be willing to use AI (adjusted odds ratio (AOR) = 3.647; 95% confidence interval (CI): 2.204-6.036; p < 0.001). A positive perception also significantly predicted willingness (AOR = 5.442; 95% CI: 3.248-9.118; p < 0.001). Age and gender were not significant predictors.

Conclusion

Undergraduate health science students demonstrated a high level of knowledge, positive perceptions, and a high willingness to use AI for personalized health recommendations. Knowledge and perception were significant factors associated with willingness, emphasizing the importance of integrating AI-related education into health-science curricula to support digital transformation in healthcare.

## Introduction

Artificial intelligence (AI) refers to the development of machines capable of mimicking human cognitive functions such as thinking, learning, and problem-solving [1]. Over the past several decades, technological development has significantly transformed healthcare delivery. AI has a growing role in supporting diagnostic processes, drug development, health data analysis, and personalized health recommendations [2]. Personalized medicine aims to tailor diagnostics and interventions to individuals based on their genetic, molecular, physiological, behavioral, and environmental characteristics [3]. AI enhances this process by identifying complex patterns in large datasets and predicting treatment responses, ultimately supporting more precise clinical decision-making [2,4,5].

AI has been applied to support early diagnosis, risk stratification, individualized treatment planning, and disease prevention. It also contributes to clinical workflow optimization through application to electronic health records (EHRs), automated decision support, and robotic-assisted procedures [6,7]. Evidence also indicates variability in trust and acceptance of AI-based health solutions across settings. For example, a German study found overwhelming support for AI-assisted early detection of skin cancer, particularly among high-risk individuals [8]. However, a study in China highlighted that although patients valued AI, 91.3% still preferred physician judgment when discrepancies occurred between human and AI recommendations [9]. These findings indicate differing levels of trust in AI across populations.

In the Saudi Arabian context, a limited awareness of personalized medicine has been reported among healthcare professionals, with 68.75% demonstrating inadequate knowledge despite a strong interest in additional training and understanding of individual disease risks [10]. This highlights the importance of examining AI-related knowledge and perceptions in the local context, particularly among future healthcare professionals.

Several technology-adoption frameworks (e.g., Theory of Planned Behavior (TPB), Technology Acceptance Model (TAM), and Unified Theory of Acceptance and Use of Technology (UTAUT)) broadly emphasize the role of knowledge-related factors and perceptions in shaping behavioural intention to use new technologies [11-13]. In this study, we focus on students’ self-reported knowledge, perceptions, and willingness to use AI-based personalized health recommendations to inform future educational planning and digital transformation efforts in healthcare. Undergraduate health students represent the future healthcare workforce; therefore, assessing their knowledge, perceptions, and willingness to use AI may inform training priorities and capacity-building efforts in healthcare systems. For conceptual clarity in this study, readiness is defined as students' perceived preparedness and capacity to engage with AI-based personalized health recommendations, whereas willingness is defined as their self-reported openness and intention to use such tools.

This study specifically aimed to: (1) determine the level of knowledge about AI among undergraduate students; (2) assess their perceptions of AI; (3) determine their willingness to use AI for personalized health recommendations; and (4) examine whether significant associations exist between knowledge, perception, and willingness to use AI.

The following hypotheses were tested: (1) students with a high level of knowledge about AI are more likely to use AI for personalized health recommendations; and (2) there is a significant relationship between the level of knowledge, perception, and willingness to use AI for personalized health recommendations.

## Materials and methods

Study design

A descriptive cross-sectional study design was used to assess the level of knowledge, perception, and willingness to use AI for personalized health recommendations among undergraduate students in health colleges at King Faisal University.

Study setting and population

The study was conducted among male and female undergraduate students enrolled in the health colleges at King Faisal University. All students who were enrolled during the data collection period and met the inclusion criteria were eligible to participate.

Students were included if they were: currently enrolled in one of the health colleges, and willing to voluntarily participate in the study. Students were excluded if they were not enrolled as undergraduate students at the time of data collection or provided incomplete responses to essential parts of the questionnaire.

Sample size and sampling technique

Sample size was estimated using OpenEpi (https://www.openepi.com/Menu/OE_Menu.htm) for a cross-sectional study design, assuming a 95% confidence level, 5% margin of error, design effect of 1.0, and an expected proportion of 47% based on a previous study [14]. The minimum required sample size was 358 participants. To account for potential non-response, 10% was added (target sample = 394). The final analyzed sample included 417 students. The sample size estimation was intended to provide adequate precision for key proportions in a cross-sectional survey. Participants were recruited using a convenience sampling approach from eligible undergraduate health students at King Faisal University.

Data collection tool

Data were collected using a structured, self-administered electronic questionnaire adapted from previously published studies assessing students’ knowledge, perceptions, and attitudes toward AI in healthcare and medical education, including the framework originally described by Sit et al. [15] and subsequently adapted in regional studies [16,17]. The questionnaire was adapted to align with the objectives and context of the present study and was piloted prior to data collection to assess clarity and comprehensibility; no modifications were required following the pilot. The final questionnaire comprised sections on sociodemographic characteristics, knowledge of AI, perceptions of AI, and willingness to use AI in personalized health recommendations. As the questionnaire was adapted for the present study, the final version used in this sample was not treated as a formally validated psychometric scale; therefore, the findings should be interpreted with this consideration in mind.

Data collection procedure

Data were collected electronically using a self-administered online questionnaire distributed to eligible undergraduate health students at King Faisal University between October and November 2024. Participation was voluntary, and informed consent was obtained electronically before questionnaire completion. The questionnaire link was shared through student communication channels (including student groups on social media platforms). Because the survey link was disseminated broadly through group channels, the total number of students who received or viewed the link could not be determined; therefore, a precise response rate could not be calculated. Responses were screened for completeness before analysis.

Ethical considerations

Ethical approval for this study was obtained from the King Faisal University Institutional Review Board (IRB) (Approval No. KFU-2024-ETHICS2489). Participation was anonymous, voluntary, and no personal or identifying information was collected. Students were informed of the study objectives, their right to withdraw at any time, and assured of confidentiality.

Data analysis

Data were analyzed using JASP (Version 0.95.4; University of Amsterdam, Amsterdam, The Netherlands). Descriptive statistics, including frequencies, percentages, means, and standard deviations, were used to summarize participants’ characteristics and domain scores (knowledge, perceptions, and willingness). For descriptive classification, domain scores were categorized using study-specific cutoffs. Knowledge and perception scores were categorized using the sample mean as the cutoff (knowledge mean = 3.3; perception mean = 29.5), and willingness was categorized using a cutoff score of 2.9. These cutoffs were used for descriptive reporting only and do not represent validated thresholds. Logistic regression analysis was performed to examine the associations between knowledge, perception, and willingness to use AI for personalized health recommendations. Statistical significance was set at p < 0.05.

## Results

Participant characteristics

A total of 417 undergraduate students participated in the study. Most participants were aged 20-21 years (n = 169, 40.5%), followed by 22-23 years (n = 115, 27.6%), 19 years or younger (n = 113, 27.1%), and 24 years or older (n = 20, 4.8%). Participants were drawn from several health colleges, primarily the College of Medicine (n = 141, 33.8%) and the College of Applied Medical Sciences (n = 129, 30.9%) (Table 1).

**Table 1:** Sociodemographic characteristics of participating undergraduate students (n = 417)

Variable	n	Percent
College of Medicine	141	33.8%
College of Applied Medical Sciences	129	30.9%
College of Veterinary Medicine	72	17.3%
College of Clinical Pharmacy	51	12.2%
College of Dentistry	24	5.8%
Female Gender	236	56.6%
Male Gender	181	43.4%
Age 19 or below	113	27.1%
Age 20–21	169	40.5%
Age 22–23	115	27.6%
Age 24 or above	20	4.8%

Level of knowledge about AI

Most students demonstrated a high level of knowledge about AI, accounting for 67.87% (95% CI: 63.24-72.17), while 32.13% had a low level of knowledge. “High/low knowledge” and “positive/negative perception” were categorized using the study-specific mean-based cutoffs described in the Methods and are presented for descriptive purposes only (Table 2).

**Table 2:** Level of knowledge about artificial intelligence among undergraduate students (n = 417)

Knowledge Level	n	Percent
High Knowledge	283	67.87%
Low Knowledge	134	32.13%

Perception toward AI

More than half of the students reported a positive perception of AI (57.07%; 95% CI: 52.28-61.74), whereas 42.93% reported a negative perception (Table 3).

**Table 3:** Perception toward artificial intelligence among undergraduate students (n = 417)

Perception Level	n	Percent
Positive Perception	238	57.07%
Negative Perception	179	42.93%

Willingness to use AI

The majority of respondents expressed willingness to use AI for personalized health recommendations (74.82%; 95% CI: 70.44-78.75). The willingness cut-off was set at a mean score of 2.9 (Table 4).

**Table 4:** Willingness to use artificial intelligence in personalized health recommendations (n = 417)

Willingness	n	Percent
Willing	312	74.82%
Not Willing	105	25.18%

Association between knowledge, perception, and willingness

Multiple logistic regression analysis was performed to examine the associations between knowledge, perception, and willingness to use AI while adjusting for age and gender (Table 5). Students with a high level of knowledge had higher odds of willingness to use AI compared to those with low knowledge (AOR = 3.647; 95% CI: 2.204-6.036; p < 0.001). Students with a positive perception had higher odds of willingness to use AI compared to those with a negative perception (AOR = 5.442; 95% CI: 3.248-9.118; p < 0.001). Age and gender were not statistically significant predictors of willingness to use AI in the adjusted model.

**Table 5:** Logistic regression analysis of factors associated with willingness to use artificial intelligence in personalized health recommendations

Variable	Category	Adjusted Odds Ratio (AOR)	95% CI	p-value
Level of knowledge	High knowledge	3.647	2.204–6.036	<0.001
	Low knowledge (reference)	1.000	-	-
Perception level	Positive perception	5.442	3.248–9.118	<0.001
	Negative perception (reference)	1.000	-	-
Gender	Male	0.880	0.532–1.455	0.617
	Female (reference)	1.000	-	-
Age group	20–21	1.050	0.558–1.974	0.881
	22–23	0.827	0.423–1.618	0.579
	24 or above	1.517	0.367–6.270	0.565
	19 or below (reference)	1.000	-	-

## Discussion

To the best of our knowledge, this is the first study conducted in Al-Ahsa to examine the relationship between knowledge, perception, and willingness to use AI for personalized health recommendations among undergraduate students. The sample of 417 students, representing 5 health colleges at King Faisal University and including both genders, provides insight into students’ knowledge, perceptions, and willingness to use AI for personalized health recommendations within the study setting.

Most participants (67.87%) demonstrated a high level of knowledge about AI, reflecting generally good awareness among health-science students. These findings align with those of Jha et al., who reported similar variations in perceptions among medical students and practitioners regarding the impact of AI on future clinical practice [18].

Although students expressed high levels of interest and generally positive perceptions, responses to some perception items suggested ongoing caution regarding the extent to which AI can replace or outperform human clinical expertise. This cautious stance is consistent with Buabbas et al., who found that while students acknowledge AI’s potential, many remain hesitant about substituting human clinical judgment with AI-generated output [17]. These attitudes reflect broader trends, suggesting that trust and perceived reliability influence the acceptability of AI in healthcare. Behavioral technology-adoption frameworks (e.g., TPB, TAM, and UTAUT) may provide general interpretive context for the observed associations, but they were not directly tested in this study.

Logistic regression analysis confirmed both knowledge and perception as predictors of willingness to use AI for personalized health recommendations. These findings are consistent with a multicenter study, conducted across 10 Arab countries and involving 1,713 nursing students, which demonstrated that improvements in knowledge and attitudes significantly increased intention to adopt AI in clinical practice [19]. Together, these findings highlight the importance of integrating AI-related competencies into health-science curricula. These findings should be interpreted as associations within a cross-sectional design and do not establish causal relationships.

This study has several limitations. The cross-sectional design precludes causal inference, and the observed associations cannot establish directionality. Data were self-reported and may be subject to response bias, including social desirability bias. The study was conducted at a single university using an online questionnaire, which may limit generalizability and introduce selection bias. In addition, although the questionnaire was adapted from previously published studies, the modified version used in this study was not treated as a formally validated psychometric scale in this sample, which may affect measurement precision and interpretation of construct-level findings. Finally, willingness to use AI was assessed as a self-reported hypothetical intention and may not directly translate into actual adoption behavior in real-world settings. Academic program and year of study were not included in the regression model and may contribute to residual confounding.

## Conclusions

This study provides insight into students’ knowledge, perceptions, and willingness to use AI for personalized health recommendations within the study setting. Overall, students demonstrated a high level of knowledge, positive perceptions, and a high willingness to engage with AI-based tools. Knowledge and perception were significant predictors of willingness to use AI, underscoring the importance of strengthening AI-related competencies within health-science education.

These findings highlight the need for the integration of AI concepts, applications, and ethical considerations into health-science curricula to better prepare future healthcare professionals for the digital transformation of healthcare. Enhancing students’ understanding of and shaping their attitudes toward AI may facilitate broader acceptance and responsible adoption of AI-enabled personalized health systems. Continued efforts to build awareness and promote training opportunities will support the effective and safe integration of AI into clinical practice and public health settings.
